# Items

**Published:** 1881-10

**Authors:** 


### Items.

THE FIFTH INTERNATIONAL PHARMACEUTICAL CONGRESS was held in London, recently. The congress adopted resolutions favoring a universal pharmacopœia, and providing for the appointment of a commission, to consist of two members from every country represented at the meeting, to carry out the objects of the resolutions.

The invitation to hold the next session of the Congress in Philadelphia was declined, and the body adjourned to meet in Brussels in 1884.

---

DR. W. G. OWEN has been elected Professor of the Principles and Practice of Medicine, and Dr. W. D. Bizzell, formerly Professor of Chemistry in the Medical College of Alabama, Mobile, has been elected to the chair of chemistry, in the Southern Medical College, Atlanta.

---

THE *Louisville Medical News* is disturbed about the quality of the ice furnished to some of the western cities. A large part of the supply is obtained from streams polluted by sewage matter, the over-flow from filthy canals, seepage from innumerable manure heaps, slaughter-pens, privies, etc. The *News* asserts the generally entertained idea that freezing purifies water is a popular fallacy, and suggests that the so-called lake ice, obtained from impure water, is a frequent source of disease.

---

DR. GEORGE H. ROHE, formerly a resident of this city, has recently been elected Clinical Professor of Dermatology in the College of Physicians and Surgeons, Baltimore. We have the pleasure of presenting in this issue the first of a series of practical papers on diseases of the skin, by Prof. Rohe.

---

THE REGISTER acknowledges the courtesy of Dr Robert Battey, which has enabled it to lay before its readers a synopsis of the proceedings of the International Medical Congress, in London; a large assemblage, which rejoiced in the presence of many great men.

---

THE AMERICAN PUBLIC HEALTH ASSOCIATION will convene in its ninth annual session, at Savannah, November 19th, next, and continue four days. A full attendance and a profitable meeting is anticipated.

---

DR. J. S. TODD, has accepted the chair of Materia Medica and Therapeutics in the Atlanta Medical College, to which he has been elected since the last session.

